# Strengthening health systems by health sector reforms

**DOI:** 10.3402/gha.v7.23568

**Published:** 2014-02-13

**Authors:** Flavia Senkubuge, Moeketsi Modisenyane, Tewabech Bishaw

**Affiliations:** 1 Health Policy and Management, School of Health Systems and Public Health, University of Pretoria, Pretoria, South Africa; 2 Alliance For Brain Gain and Innovative Development (ABIDE), Addis Ababa, Ethiopia; 3 African Federation of Public Health Associations (AFPHAs), Addis Ababa, Ethiopia; 4 Ethiopian Public Health Association (EPHA), Addis Ababa, Ethiopia

**Keywords:** health sector reforms, systems thinking, health systems, systems-level interventions, global public health, global health, efficiency, equity

## Abstract

**Background:**

The rising burden of disease and weak health systems are being compounded by the persistent economic downturn, re-emerging diseases, and violent conflicts. There is a growing recognition that the global health agenda needs to shift from an emphasis on disease-specific approaches to strengthening of health systems, including dealing with social, environmental, and economic determinants through multisectoral responses.

**Methods:**

A review and analysis of data on strengthening health sector reform and health systems was conducted. Attention was paid to the goal of health and interactions between health sector reforms and the functions of health systems. Further, we explored how these interactions contribute toward delivery of health services, equity, financial protection, and improved health.

**Findings:**

Health sector reforms cannot be developed from a single global or regional policy formula. Any reform will depend on the country's history, values and culture, and the population's expectations. Some of the emerging ingredients that need to be explored are infusion of a health systems agenda; development of a comprehensive policy package for health sector reforms; improving alignment of planning and coordination; use of reliable data; engaging ‘street level’ policy implementers; strengthening governance and leadership; and allowing a holistic and developmental approach to reforms.

**Conclusions:**

The process of reform needs a fundamental rather than merely an incremental and evolutionary change. Without radical structural and systemic changes, existing governance structures and management systems will continue to fail to address the existing health problems.

Health sector reforms can be defined as ‘sustained, purposeful changes to improve the efficiency, equity, and effectiveness of the health sector’ (1). However, health sector reform is not a concept that demands a single global definition (2, 3).

Based on the definition above, by ‘fundamental’ change, the reform should be ‘purposeful’; therefore elements and components of the reform need to have been developed in a rational manner (3, 4). Third, the reform should be ‘sustainable’. Most fundamental changes will be sustained because they involve significant transformation of systems and the creation of actors who will defend their new interests in the political process (3, 4).

There is growing evidence of the need for a paradigm shift from efficiency-directed reforms of the 1990s to gender- and equity-oriented health reforms (5, 6). The objective is to ‘increase the efficiency and effectiveness with which health systems reach the poor and disadvantaged’ (5). Given the complexity of health system reforms, there is a need for a more coherent approach to change that includes a deeper understanding of the contexts of reforms; understanding how the health system operates; the need for information for decision making; and institutions issues (1, 4, 7). On the contrary, it is imperative to understand the health system, whose goals are ‘improving health and health equity in ways that are responsive, financially fair, and make the best or most efficient use of available resources’ (8). The health system is therefore ‘more than a pyramid of public-owned facilities that deliver personal health services’ and includes state and non-state actors such as non-governmental organizations, civil society organizations, and the private sector (8). The WHO health systems framework consists of six building blocks, namely, service delivery, health workforce; health information; medical technologies (including medical products, vaccines, and other technologies); health financing; leadership; and governance (8, 9).

These building blocks alone do not constitute a system. It is the multiple relationships and interactions among the blocks that convert these blocks into a system. Health systems are then a dynamo of interactions, synergies, and shifting sub-systems (10, 11). Furthermore, it is important to highlight the role of people, not just as mediators and beneficiaries, but also as actors in driving the system itself (11). This includes their participation as individuals, civil society organizations, stakeholder networks, and key actors influencing each of the building blocks, as health workers, managers, and policy makers (11, 12).

Systems thinking, on the contrary, demand a deeper understanding of the linkages, inter-relationships, interactions, and behaviors among elements that characterize the entire system (8, 13). In the context of the health sector, there is a need to shift focus to the nature of the relationships among the building blocks; the spaces between the building blocks; and the synergies emerging from interactions among the blocks (8, 12).

In this paper, we propose a framework to understand the interrelationships connecting health sector reform processes and health systems strengthening, with specific focus on systems thinking and on interactions between health reforms and country health systems. The analysis of the interrelationships between these factors provides a useful tool to predict the effects of different ‘technical designs’ on the elements of health systems that they affect.

## Framework and methods

The paper reviewed available literature using a conceptual framework that was adapted from two existing models – one that identifies distinct functions or building blocks of health systems and the other that describes the interactions between health sector reforms and these functions (8, 9).

**Fig. 1:**
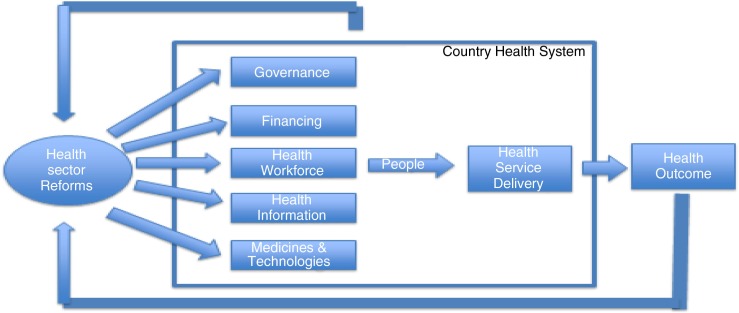
Conceptual framework of the interaction between health sector reforms and country health system.

In our conceptual framework, we identified five points of interactions between health sector reforms and country health systems, namely, governance, finance, health workforce, health information systems, and supply management systems. We then explored how these five points interlink and contribute toward the sixth point of interaction, namely, the delivery of health services. The proposed framework is represented in Fig. 1. The central role of people is recognized in our model. Also, all aspects of the six points of interaction take place within a general context that includes economic, social, political, environmental, and other factors that are not included in our analysis. The conceptual framework data and analysis have limitations that arise because health systems are ‘complex adaptive systems’ (13, 14).

## Findings

### Complex health challenges

Around three quarters of the world's absolute poor live in middle-income countries and many of the world's poorest people will remain dependent on external financial and technical support (15). This dependence raises important questions about how the health systems, especially those of the developing countries, should be transformed and financed. The 21st century has also seen a transformation in the relative power of the state on one hand and markets, civil society, and social networks of individuals on the other hand. There is a growing recognition of the role of non-state actors, such as the private sector, as an engine of growth and innovation, and also in the delivery of health services (16). Foreign direct investment and remittances far outstrip development support in many low-income countries (4). Individuals and civil society have been empowered on a scale that was not foreseen at the beginning of the last decade (17).

Beyond their epidemiological and demographic aspects and availability of a range of interventions, new political, economic, social, and environmental realities present another more complex agenda for global health. With growing complexity comes the need for a greater focus on the *means* by which better health outcomes can be secured. This includes health as a human right; health equity; stronger and more resilient health systems; health in all policies; and technological innovation and efficiency in the face of financial constraints. The new health agenda needs to acknowledge the close links between health and sustainable development (18, 19).

Indeed, health governance is no longer the exclusive preserve of nation states. Collaborative governance is the new order that fosters and promotes the working together of non-health actors and health actors (20). Civil society networks, individual non-governmental organizations at international and community levels, professional groups, philanthropic foundations, trade associations, the media, national and transnational corporations, individuals, and informal diffuse communities have found a new voice and influence in reforming the health sector (21).

The potential and actual impact of health sector reform on health systems has been highlighted in several studies (1–4). These reforms were usually embedded in a set of government reforms intended to improve the efficiency, equity of access, and quality of public services in general. We argue that using a systemic thinking approach and understanding the systemic factors and their effects is important in assessing whether reforms can effectively reach their goals. Each element of the health system can block technically well-designed health reforms and consequently deprive populations of their potential benefits. Without this broad understanding of a systems’ capacity, researchers and policy designers in many countries fail to design health sector reforms and specific interventions that optimizes the health systems’ ability to deliver essential health interventions. There is also another poorly appreciated phenomenon, that every health intervention, such as health sector reforms, from the simplest to the most complex, has an effect (both positive and negative) on the overall system (11, 21).

### Health service delivery

The main output of any health system is to ensure the delivery of health services that are accessible, equitable, safe, and responsive to the needs of the users (8). Health service delivery is the backbone of any health system. Importantly, delivery of health services will depend on the availability of health facilities, health workers, diagnostics, drugs, and other supplies, including provision for financing and existence of responsive communities. For the purpose of this paper, we focus on three key factors, namely, service access and coverage, equity in services, and service quality.

Growing evidence shows that focusing on strengthening health systems has a positive effect on access and uptake of some services (22, 23). Further, evidence shows that many health systems interventions and reforms have led to an increase in coverage of several health services (22, 23). Importantly though, apart from a small number of studies, the interaction between health systems and health interventions is not well explored (24, 25).

One of the key objectives of many health sector reforms and health systems strengthening is to increase equity in access to health services for those in need, regardless of their social and economic status (5, 6). Data in general has shown an overall trend of improvement of equity in access and outcomes due to many health sector reforms and health systems interventions, such as ensuring that services are free at the point of delivery; engaging communities and civil society organizations in the planning and delivery of health services; and introduction of a decentralization agenda in the delivery of services (17, 26). Nevertheless, inequality in access and coverage continue to plague many countries.

The delivery of quality health services remains another important goal of most health reforms and health systems. However, there is evidence that indicates that pressure to meet numerical targets may have a detrimental effect on the quality of services (27).

### Financing

Health financing is more than a matter of raising money for health. We focus on the intersection of health sector reforms and three key factors that affect the performance of health systems financing, namely, collection of revenue, pooling of funds, and purchasing.

In many countries, especially low- and middle-income, financial protection and access to needed health care are growing priorities. In an effort to diversify domestic sources of revenue, many countries have introduced a series of health financing reforms in order to strengthen health systems (28, 29).

For example, many countries are moving toward universal health coverage which will be achieved mainly through prepayment health financing mechanisms where funds will be pooled to enable subsidies to flow from rich to poor and the healthy to sick (15). In countries such as Thailand and Moldova, this system resulted in increased access and reduction in inequity (30).

On the contrary, Mexico, about 25 years ago, had different types of pools which covered different population groups, each with different levels of benefits. This system resulted in not only an inequitable but also an inefficient and costly health system, and hence these challenges resulted in the recent health reforms in Mexico (31). Some studies have suggested that moving to a system of prepayment and pooling of funds does not necessarily guarantee access to health services. Other issues that result in reduced access to health care may be cultural barriers, language barriers, and transport costs (32, 33).

Therefore, in the absence of looking at specific policy designs and implementation features, such as institutional capacities, proper economic and financial evaluations, and risk analysis, the goal of universal coverage in many low- and middle-income countries may be a challenge. In purchasing health services, many countries have implemented a number of reforms, such as a capitation system and a case-based payment system (15). However, both the capitation system and case-based payment system require the ability to measure the costs accurately before they are implemented and to monitor their impact over time.

Therefore, a mixed payment system like that adopted in Thailand may be explored, including establishing quality monitoring for providers’ behavior. Taiwan's bold legislative act of a single-payer, national health insurance scheme is another consideration (34). Pay-for-performance, emerging as an innovative and efficient mechanism in the US and UK, should also be explored in combination with other methods to improve quality of care (35).

### Governance and leadership

According to the conceptual framework of governance by Brinkerhoff and Bossert (36), health governance involves three main sets of actors, namely state actors, health service providers and health service users, and the general public (36). Therefore, effective health system governance – engaging and regulating both public and private sector actors – is crucial for achieving broader health objectives (37). We focus on the intersection between health sector reforms and three key factors that affect the performance of health systems governance, namely, policies and provision of oversight; stakeholder participation; and health system responsiveness, accountability, and regulation.

Good governance has become a priority public health agenda (38). Good governance in a country directly affects the environment in which the health system operates and health officials exercise their responsibilities. Measures of overall governance include voice and accountability, political stability, government effectiveness, regulatory quality, rule of law, and control of corruption (39).

The effectiveness and quality of linkages between state, citizens, and providers influences the ability of the health system to meet the performance criteria measures of equity, efficiency, access, quality, and sustainability (36). Inclusion of civil society ideas into policy development shows both the strength of civil society in being a reliable partner to government, as well as government willingness to listen to civil society concerns (40). Other studies have also highlighted the important contribution of global health initiatives to the funding of health (41) Furthermore, in many developing countries there has been movement to strengthen participation of health care users in decision making as part of the health sector reforms and health systems strengthening (17, 42).

Although some successful efforts to include non-state actors are identified, these have to be closely monitored and evaluated. Careful judgments have to be made concerning the relative return on investment in improving non-state actors’ activities as opposed to investment in a strengthened public sector (27).

### Health workforce

Human resources for health are the foundation of the health care system. The health workforce ‘works in ways that are responsive, fair, and efficient to achieve the best health outcomes possible, given available resources and circumstances’ (8). WHO has estimated the global deficit of trained health workers to be more than 4 million (43). We focus on the interaction between health sector reforms and three key factors that indicate the expected performance of the health workforce function of the health system, namely production; distribution; and retention of health workers.

Shortage of human resources for health has been reported as the main barrier to scale-up health systems and health specific interventions (44). Health systems are becoming more complex and costly, professionals are encountering more socially diverse patients with chronic conditions, patient management now requires teamwork, and there is an explosive growth of knowledge and technologies, all placing additional demands on health workers (45). Some of the current health professional education reforms are fragmented, outdated, and with static curricula, and hence producing ill-equipped graduates from underfinanced institutions (44, 45). Education for health professionals has failed to deal with ‘complex and adaptive’ health systems because of curricula rigidities, professional silos, static pedagogy, insufficient adaptation to local contexts, and commercialism in the profession (45).

To address these shortfalls, a number of educational reforms have been initiated to develop professional competencies that are responsive to changing health needs and complex health systems. Some of the health sector reforms and health systems strengthening were recruitment of health workers; creation of a mid-level cadre; use of community health workers; changing of medical and nurse training (45, 46). Nevertheless, some of these reforms lack leadership and incentives and are impeded by the tribalism of the profession as well as weakness in the power to deliver on their promise.

For instance, low- and middle-income countries have workforce shortages, skills-mix imbalances and mal-distribution in terms of skills, targeted diseases, and geographical distribution (45). This mal-distribution is due in part to the failure of the health system to attract health care workers and to retain them once there (47). International migration of the health workforce can also not be ignored. Yearly, health workers migrate from developing to developed countries (48). This ‘brain drain’ exacerbates the poor distribution of health and financial resources in already resource constrained and disease burdened health systems (48). The consequences of this migration have impacted poor countries in such a way that health systems have been weakened, there has been failure to provide much needed public health interventions and financial loses have been significant (48, 49). There is an urgent need for countries to have a system that targets motivations for global health worker migration at both individual and system level (50). An urgent relocation of resources is needed where sharing occurs between developed and developing countries so as to respond to needs of countries that are most affected (50). A number of governments have implemented a series of reforms to improve the distribution of health workers in rural and remote areas, through introduction of incentives such as allowances for housing, transportation, hardship, and education (47).

Furthermore, other global health initiatives, such as The Global Fund, have recently provided additional financial incentives to improve working and living conditions (51). However, while financial incentives may be important determinants of worker motivation, they alone cannot resolve and have not resolved all worker motivation problems. Therefore, there is a need to relook at workforce migration and also to radically reform health education in view of the opportunities for learning and joint solutions offered by globalization. Health workforce reforms should use systems thinking to strengthen health systems, including identifying organizational and cultural values that might facilitate or impede reform implementation.

### Health information system

Well-functioning health information systems will provide a better understanding of the interaction of health sector reforms and country health systems. We focus on the interaction between health sector reforms and three key factors that indicate performance of health information systems, namely, availability and accuracy of the system; use and demand of information; and innovation.

Availability and accuracy of data is a challenge, especially in many low- and middle-income countries. The challenge is specifically related to coordination and leadership; lack of adequately trained human resource at all levels and of government investment in the processes for the production of health information; and infrastructure (52). There is broad consensus that improved health outcomes can only be achieved by strengthening health systems (including health information systems) as a whole, rather than focusing on discrete, disease-focused components (53). Hence, a number of governments have implemented a series of health sector reforms to improve availability and accuracy of data to permit adequate monitoring of progress. For instance, Taiwan makes use of linked functions in a network that allows a select set of professionals access to its database (54). Further, there are emerging innovations in the generation and use of information systems even in low-income countries (55).

Of note though is that, despite efforts by governments toward harmonization and alignment of health information systems, stakeholders such as developmental partners, non-governmental organizations, and the private sector continue to pursue the development of stand-alone information systems independent of the country health information system (21). Each project or initiative has then a new, small, and un-sustainable health information system set up every time, and this hampers the development of one information system for the region or state health system.

### Supply management system

According to WHO, ‘a well-functioning health system ensures equitable access to essential medical products, vaccines, and technologies of assured quality, safety, efficacy and cost-effectiveness, and their scientifically sound and cost-effective use’ (8). Hence, uninterrupted supplies of essential health commodities and technologies are necessary. We focus on the interaction between health sector reforms and two key factors for the management of supply chain system, namely, procurement and distribution, and quality.

In many countries, there is an increase in demand for drugs, vaccines, diagnostics and laboratory materials of good quality. Many governments have developed their national capacities in procurement to respond to this increasing demand. A number of procurement and distribution reforms in many countries have resulted in a reduction in the price of some drugs, particularly those relating to the treatment of HIV/AIDS and tuberculosis (56).

Although a number of global health initiatives have assisted governments in strengthening their supply chain management systems, there is evidence that indicates instances of duplication leading to increased operational costs (21). Furthermore, poor planning and coordination has resulted in some categories of products being out of stock or overstocked. However, there are some initiatives taken to improve coordination and align procurement and distribution within government systems.

To improve quality, many governments work with WHO to increase access to good quality drugs, vaccines, health technologies, and commodities. Improvements have been noted in several countries due to the establishment of mechanisms such as drug facilities and the WHO/UN Prequalification program (57). Therefore, supply chain management systems reforms should ensure alignment with national procurement systems. Country supply management systems also need to be supported by adequate logistics information systems and a qualified health workforce.

## Conclusion

There is a need for governments to increase commitment and investment in strengthening health systems. In strengthening the health sector, national health systems stewards need to ensure alignment and coherence of policies, priorities amongst different stakeholders; manage and coordinate partnerships and expectations; and implement and foster ownership of health systems interventions at national and subnational levels. There is also a need to ensure equity gender and other aspects with regard to the provision of health services, with particular attention given to women, children, and other disadvantaged and vulnerable groups.
